# Supplementary material for: Parallel but distinct adaptive routes in the budding and fission yeasts after 10,000 generations of experimental evolution
Source: Nat Ecol Evol. 2026 Mar 13;10(4):765–78. doi: 10.1038/s41559-026-03017-1 (PMC13076206; doi:10.1038/s41559-026-03017-1)
Supplement: Supplementary file 1 — Supplementary Tables 1 and 3 and references. [file 41559_2026_3017_MOESM1_ESM.pdf]

nature ecology & evolution

Supplementary information https://doi.org/10.1038/s41559-026-03017-1

Parallel but distinct adaptive routes in the budding and fission yeasts after 10,000 generations of experimental evolution

In the format provided by the authors and unedited

**Gene symbol/ systematic ID (Number of populations with a hit (PH))**	**Function(s) (biological process)**	**Gene category (Link with the response to HS and OS)**	**Characterization of the hit(s) (Gene product truncation or potential LOF)**
bmc1/ SPBC2A9.10 (2)	Bin3 family RNA methyltransferase (snRNA metabolic process)	Others	• 1 frameshift insertion in both G1 and G3 (Truncation of 14 out of 268 residues)
bop1/ SPAP32A8.03c (2)	ubiquitin-protein ligase E3 (protein degradation)	HS-related (Ubiquitin-dependent protein degradation is ATP-dependent[1,2]. The response to HS requires specific E3 ligases that activate sterol regulatory elements (SRE) and the decrease of the activity of other E3 ligases that do not contribute to the HS response, e.g. Ubr1[3–5])	• 1 frameshift insertion in both G11 and H12 (Truncation of 475 out of 513 residues)
clg1/ SPBC1D7.03 (2)	Cyclin-like protein involved in autophagy (signaling, molecular activity regulation and autophagy)	HS- and OS-related (in *S. pombe*, *clg1* regulates autophagy[6]. Autophagy plays an important role in the response to HS and OS by recycling biomolecules	• 1 indel in the 5'UTR of the populations G2 and G3

		and removing damaged cell components[7,8])	
cmr2/ SPAC56F8.02 (2)	acetyl-CoA biosynthesis (respiration)	HS- and OS-related (Acteyl-CoA is involved in the first step of the TCA cycle and respiration enzymes are involved in energy production and ROS removal[9])	• 1 missense mutation in the AMP-dependent synthetase/ligase domain[10] in G1<br>• 1 nonsense SNP in H11 (Truncation of 174 amino acid residues in the Dip2-like domain[10] out of 1517 protein residues)
ctf18/ SPBC902.02c (4)	Ctf18 RFC-like complex subunit Ctf18 (DNA replication)	HS-related (Upregulated under HS[7])	• 1 indel in the first intron of the gene in the populations G3, G5, H9, and H11
dep1/ SPBC21C3.02c (2)	Rpd3L histone deacetylase complex subunit (chromatin organization and transcription regulation)	HS-related (Upregulated under HS[7]. *dep1Δ* strain is more sensitive to hydrogen peroxide[11] than WT)	• 1 indel in 3'UTR in the populations G2 and G11
fet4/ SPBP26C9.03c (2)	plasma membrane iron/zinc ion transmembrane transporter (inorganic ion homeostasis and transmembrane transport)	HS- and OS-related (Iron cofactors support major biochemical processes like the heme biosynthesis, TCA cycle, electron transport chain, translation, and DNA repair[12])	• 1 indel in 5'UTR in the populations G2 and H11
fhn1/ SPBC1685.13	Eisosome assembly protein	HS-related (Upregulated	• 1 indel in 3'UTR in the populations G3 and H11.

(3)	(membrane organization)	during the response to hydrogen peroxide[13])	1 indel in 3'UTR in the populations G9
iss9/ SPBC2A9.11c (2)	SAC3/GANP/THP3 family protein (transcription or RNA surveillance)	Others	• 1 frameshift insertion in the populations G1 and G5 (Truncation of 315 out of 395 residues)
pfl2 SPAPB15E9.01c (3)	cell surface glycoprotein, flocculin Pfl2 (cell adhesion)	HS- and OS-related (Flocculation and adhesion enhance survival by protecting against stressful conditions[14,15])	• 5 missense hits in G3: chrI_A3989282G (R->G), chrI_G3989285A (A->T), chrI_G3989294A (G->S), chrI_G3989384A (G->S), chrI_G3989390A (A->T) 4 missense hits in G5: chrI_G3989877C (R->T), chrI_G3989879A (G->R), chrI_A3989878T (R->S), chrI_G3989877C (R->T)<br>*Pfl2 is a disordered protein so it has many S, R and T residues[10,16]<br>• 2 indels in 5'UTR in the populations G1 and G5
pfl5/ SPBC1289.15 (3)	cell surface glycoprotein**,** flocculin Pfl5 (cell adhesion)	HS- and OS-related (Flocculation and adhesion enhance survival by protecting against stressful conditions[14,15])	• 2 Y→F missense hits in G2 and H12 around the middle of the protein. 1 K→E missense hit in G5<br>• The same frameshift insertion in the populations G2 and H12 (Truncation of 518 out of 1283 residues around the middle of the protein). 1 deletion in G2 (Truncation of 528 out of 1283 residues around the middle of the protein)
pof15/ SPAPB1A10.14 (2)	Ubiquitin complex adaptor; F-box protein (protein degradation)	HS-related (Downregulated under HS[7,17]. Ubiquitin-dependent	• 1 N→D missense hit in G5<br>• 1 frameshift deletion in the population H12 (Truncation of 135 out of 243 residues)

		protein degradation is ATP-dependent [1,2])	
pqr1/ SPAC6B12.07c (3)	SPX-RING-type ubiquitin-protein ligase regulating phosphate transport and homeostasis (protein degradation and transmembrane transport)	HS-related (Upregulated during the response to hydrogen peroxide[13]. Excess of intracellular phosphate and polyphosphate is associated with improper autophagy-dependent proteolysis in vacuoles[18])	• 1 SNV in 3'UTR in G9, G11 and H11
prr1/ SPAC8C9.14 (2)	stress-responsive DNA-binding transcription factor Prr1 (transcription regulation and response to oxidative stress)	OS-related (*prr1* is a positive regulator of the OS response. It binds *pap1* as part of a positive regulation of transcription, and it regulates *ctt1*, *srx1*, *trr1* during the cellular response to OS[19,20])	• 1 P→Q missense hit in the signal transduction response regulator/receiver domain in H12<br>• 1 frameshift insertion in the population H11 (Truncation of 517 out of 540 residues)
pyk1/ SPAC4H3.10c (5)	pyruvate kinase (carbohydrate metabolic process)	HS- and OS-related (The gene product catalyzes the last step of glycolysis. This influences the fermentation-respiration balance and thus	• 1 missense hit V→L in the barrel domain in H11<br>• 2 SNVs in 3' UTR in G1<br>• 1 SNV in 3' UTR in H12<br>• 1 deletion in the promoter (5' UTR) in G3<br>• 1 deletion in 5' UTR in G4

		biomass production and stress resistance[21])	
pzh1/ SPAC57A7.08 (2)	serine/threonine protein phosphatase (signaling)	HS- and OS-related (*pzh1* is a positive regulator of the response to OS[22]. It is also upregulated under HS[7])	• 1 deletion of 2 residues (positions 73 and 74) in the N-terminal disordered region[10] in both G5 and G9
rpp1/ SPAC3A12.04c (2)	RNase P and RNase MRP subunit p30 (ribosome biogenesis and tRNA metabolic process)	Others	• 1 SNV in the fourth intron of the gene in populations H11 and H12
seb1/ SPAC222.09 (2)	poly(A)site selection protein (mRNA and snoRNA metabolic process)	Others	• 1 missense T→I hit in the RNA recognition motif domain in G1<br>• 1 missense K→N hit in the CTD-interacting domain in G2
ubp9/ SPBC1703.12 (2)	ubiquitin C-terminal hydrolase Ubp9 (protein degradation and vesicle-mediated transport)	HS- and OS-related (*ubp9Δ* strain is more sensitive to hydrogen peroxide[11]. Protein degradation and vesicle-mediated transport enable the recycling of biomolecules under stress conditions[1,2])	• 1 frameshift insertion in the populations G3 and G9 (Truncation of 265 out of 585 residues, including most of the ubiquitin carboxyl-terminal hydrolase domain)

Supplementary Table 1 Multi-hit genes and their LOF mutations. The gene annotations are based on Pombase data. Gene names are represented in italics.

Filter name	Criteria
Mapping quality	Mapping quality score ≥ 15
Indels in low-complexity regions (short tandem repeats or homopolymers)	-Systematically eliminate the ones that are not supported by multiple timepoints<br>-For the recurrent ones, because they could reveal a signal of selection, they were manually investigated in IGV to make sure there are no mapping mistakes, and multiple reads in both strands should support them. We also ensured that they were not introduced or fixed at the same time across multiple populations or systematically with the same group of alleles (Fig. 3), which could indicate contamination.
Read bases quality	≥ Q20
Allele frequency	≥ 0.05
Coverage and supporting reads	Coverage ≥ 5 and at least one read in both strands support the variant (so at least 2 supporting reads in total, and always 2 supporting strands)
Contamination	Because the *S. pombe* populations were grown in parallel with populations from other yeast species (*C. glabrata* CBS138, *K. lactis* NRRL-Y-1140, *P. pastoris* EAMORG09, *S. cerevisiae* YJM978, *S. paradoxus* IFO1804; Fig. 1), we made sure that the reads we recovered from sequencing best map to *S. pombe*
Evidence for sub-clonality and linkage disequilibrium	|Pearson r| ≥ 0.9 with at least one mutation from the same population
Variation in non-fixed variant allele frequency	≥ 0.1
Initial allele frequency	Eliminate variants with an allele frequency ≥ 0.4 in at least a third of the populations

Supplementary Table 3 List of variant calling filters. Species names are represented in italics.

Supplementary References

1. Dupré, S., Urban-Grimal, D. & Haguenauer-Tsapis, R. Ubiquitin and endocytic internalization in yeast and animal cells. *Biochimica et Biophysica Acta (BBA) - Molecular Cell Research* **1695**, 89–111 (2004).
2. Zattas, D. & Hochstrasser, M. Ubiquitin-dependent Protein Degradation at the Yeast Endoplasmic Reticulum and Nuclear Envelope. *Crit Rev Biochem Mol Biol* **50**, 1–17 (2015).
3. Lee, C.-Y. S., Yeh, T.-L., Hughes, B. T. & Espenshade, P. J. Regulation of the Sre1 hypoxic transcription factor by oxygen-dependent control of DNA binding. *Mol Cell* **44**, 225–234 (2011).
4. Hughes, B. T. & Espenshade, P. J. Oxygen-regulated degradation of fission yeast SREBP by Ofd1, a prolyl hydroxylase family member. *EMBO J* **27**, 1491–1501 (2008).
5. Porter, J. R., Lee, C.-Y. S., Espenshade, P. J. & Iglesias, P. A. Regulation of SREBP during hypoxia requires Ofd1-mediated control of both DNA bindingand degradation. *Mol Biol Cell* **23**, 3764–3774 (2012).
6. Matsuda, S., Kikkawa, U., Uda, H. & Nakashima, A. The S. pombe CDK5 ortholog Pef1 regulates sexual differentiation through control of the TORC1 pathway and autophagy. *Journal of Cell Science* **133**, jcs247817 (2020).
7. Todd, B. L., Stewart, E. V., Burg, J. S., Hughes, A. L. & Espenshade, P. J. Sterol Regulatory Element Binding Protein Is a Principal Regulator of Anaerobic Gene Expression in Fission Yeast. *Mol Cell Biol* **26**, 2817–2831 (2006).
8. Farrugia, G. & Balzan, R. Oxidative Stress and Programmed Cell Death in Yeast. *Front. Oncol.* **2**, (2012).
9. Kaino, T., Tonoko, K., Mochizuki, S., Takashima, Y. & Kawamukai, M. Schizosaccharomyces japonicus has low levels of CoQ10 synthesis, respiration deficiency,

and efficient ethanol production. *Bioscience, Biotechnology, and Biochemistry* **82**, 1031–1042 (2018).

10. Harris, M. A. *et al.* Fission stories: using PomBase to understand Schizosaccharomyces pombe biology. *Genetics* **220**, iyab222 (2022).
11. Rodríguez-López, M. *et al.* Broad functional profiling of fission yeast proteins using phenomics and machine learning. *eLife* **12**, RP88229 (2023).
12. Philpott, C. C., Leidgens, S. & Frey, A. G. Metabolic remodeling in iron-deficient fungi. *Biochimica et Biophysica Acta (BBA) - Molecular Cell Research* **1823**, 1509–1520 (2012).
13. Rubio, A., Ghosh, S., Mülleder, M., Ralser, M. & Mata, J. Ribosome profiling reveals ribosome stalling on tryptophan codons and ribosome queuing upon oxidative stress in fission yeast. *Nucleic Acids Research* **49**, 383–399 (2021).
14. Kwon, E.-J. G. *et al.* Deciphering the Transcriptional-Regulatory Network of Flocculation in Schizosaccharomyces pombe. *PLoS Genet* **8**, e1003104 (2012).
15. Stewart, G. G. Yeast Flocculation—Sedimentation and Flotation. *Fermentation* **4**, 28 (2018).
16. Dyson, H. J. & Wright, P. E. Intrinsically unstructured proteins and their functions. *Nat Rev Mol Cell Biol* **6**, 197–208 (2005).
17. Chen, D. *et al.* Global Transcriptional Responses of Fission Yeast to Environmental Stress. *Mol Biol Cell* **14**, 214–229 (2003).
18. Sawada, N., Ueno, S. & Takeda, K. Regulation of inorganic polyphosphate is required for proper vacuolar proteolysis in fission yeast. *J Biol Chem* **297**, 100891 (2021).
19. Calvo, I. A., García, P., Ayté, J. & Hidalgo, E. The transcription factors Pap1 and Prr1 collaborate to activate antioxidant, but not drug tolerance, genes in response to H2O2. *Nucleic Acids Res* **40**, 4816–4824 (2012).

20. Quinn, J. *et al.* Two-Component Mediated Peroxide Sensing and Signal Transduction in Fission Yeast. *Antioxidants & Redox Signaling* **15**, 153–165 (2011).

21. Kamrad, S. *et al.* Pyruvate kinase variant of fission yeast tunes carbon metabolism, cell regulation, growth and stress resistance. *Mol Syst Biol* **16**, e9270 (2020).

22. Leiter, É. *et al.* Protein phosphatase Z modulates oxidative stress response in fungi. *Fungal Genet Biol* **49**, 708–716 (2012).
