# Supplementary material for: Parallel but distinct adaptive routes in the budding and fission yeasts after 10,000 generations of experimental evolution
Source: Nat Ecol Evol. 2026 Mar 13;10(4):765–78. doi: 10.1038/s41559-026-03017-1 (PMC13076206; doi:10.1038/s41559-026-03017-1)
Supplement: Supplementary file 2 — Reporting Summary [file 41559_2026_3017_MOESM2_ESM.pdf]

nature portfolio

Corresponding author(s): Alex N. Nguyen Ba

Last updated by author(s): Jan 22, 2026

Reporting Summary

Nature Portfolio wishes to improve the reproducibility of the work that we publish. This form provides structure for consistency and transparency in reporting. For further information on Nature Portfolio policies, see our Editorial Policies and the Editorial Policy Checklist.

Statistics

For all statistical analyses, confirm that the following items are present in the figure legend, table legend, main text, or Methods section.

n/a	Confirmed	
☐	☒	The exact sample size (*n*) for each experimental group/condition, given as a discrete number and unit of measurement
☐	☒	A statement on whether measurements were taken from distinct samples or whether the same sample was measured repeatedly
☐	☒	The statistical test(s) used AND whether they are one- or two-sided<br>*Only common tests should be described solely by name; describe more complex techniques in the Methods section.*
☐	☒	A description of all covariates tested
☐	☒	A description of any assumptions or corrections, such as tests of normality and adjustment for multiple comparisons
☐	☒	A full description of the statistical parameters including central tendency (e.g. means) or other basic estimates (e.g. regression coefficient) AND variation (e.g. standard deviation) or associated estimates of uncertainty (e.g. confidence intervals)
☐	☒	For null hypothesis testing, the test statistic (e.g. *F*, *t*, *r*) with confidence intervals, effect sizes, degrees of freedom and *P* value noted<br>*Give P values as exact values whenever suitable.*
☒	☐	For Bayesian analysis, information on the choice of priors and Markov chain Monte Carlo settings
☐	☒	For hierarchical and complex designs, identification of the appropriate level for tests and full reporting of outcomes
☐	☒	Estimates of effect sizes (e.g. Cohen's *d*, Pearson's *r*), indicating how they were calculated

Our web collection on statistics for biologists contains articles on many of the points above.

Software and code

Policy information about availability of computer code

Data collection	All available softwares used were described in the methods along with the version: Varscan2.4.6, fastp v 0.23.1, samtools v1.17, picard v2.26.3, subread v2.0.6, DESeq2 v1.34.0, drc v3.0-1, growthcurver v0.3.1, vegan (2.7-2), CytExpert (2.5)
Data analysis	The variant calling and RNA-seq analysis pipeline is available at https://github.com/arnaud00013/Experimental_evolution_S_pombe

For manuscripts utilizing custom algorithms or software that are central to the research but not yet described in published literature, software must be made available to editors and reviewers. We strongly encourage code deposition in a community repository (e.g. GitHub). See the Nature Portfolio guidelines for submitting code & software for further information.

Data

Policy information about availability of data

All manuscripts must include a data availability statement. This statement should provide the following information, where applicable:

- Accession codes, unique identifiers, or web links for publicly available datasets
- A description of any restrictions on data availability
- For clinical datasets or third party data, please ensure that the statement adheres to our policy

Raw DNA and RNA sequences are available on NCBI: https://www.ncbi.nlm.nih.gov/bioproject/PRJNA1315931. There are no restrictions on data availability.

Research involving human participants, their data, or biological material

Policy information about studies with human participants or human data. See also policy information about sex, gender (identity/presentation), and sexual orientation and race, ethnicity and racism.

Reporting on sex and gender	Sex and gender are not relevant to this study as it studies yeast populations and not human populations.
Reporting on race, ethnicity, or other socially relevant groupings	Race, ethnicity, or other socially relevant groupings are not relevant to this study as it studies yeast populations and not human populations.
Population characteristics	See above.
Recruitment	See above.
Ethics oversight	See above.

Note that full information on the approval of the study protocol must also be provided in the manuscript.

Field-specific reporting

Please select the one below that is the best fit for your research. If you are not sure, read the appropriate sections before making your selection.

☒ Life sciences ☐ Behavioural & social sciences ☐ Ecological, evolutionary & environmental sciences

For a reference copy of the document with all sections, see nature.com/documents/nr-reporting-summary-flat.pdf

Life sciences study design

All studies must disclose on these points even when the disclosure is negative.

Sample size	Sample size was determined based on the standards in the field. The Lenski LTEE had 12 replicates, which has been found to be sufficient to analyze several aspects of evolution.
Data exclusions	We excluded 5 S. pombe populations from analysis as they were contaminated or lost after 10,000 generations of evolution.
Replication	The reported study is 10 parallel populations as replication of the evolution. For DNA analysis, time-resolved sequencing allows intrinsic replication of discovered novel alleles. Phenotyping and RNA-seq was done as biological replications over different days.
Randomization	The clonal ancestors were chosen randomly from single colonies from a plate.
Blinding	Blinding to the experimental environment was not relevant to our study because the ten populations were subjected to identical data collection and data analysis procedures.

Reporting for specific materials, systems and methods

We require information from authors about some types of materials, experimental systems and methods used in many studies. Here, indicate whether each material, system or method listed is relevant to your study. If you are not sure if a list item applies to your research, read the appropriate section before selecting a response.

Materials & experimental systems

n/a	Involved in the study	
☒	☐	Antibodies
☒	☐	Eukaryotic cell lines
☒	☐	Palaeontology and archaeology
☒	☐	Animals and other organisms
☒	☐	Clinical data
☒	☐	Dual use research of concern
☒	☐	Plants

Methods

n/a	Involved in the study	
☒	☐	ChIP-seq
☒	☐	Flow cytometry
☒	☐	MRI-based neuroimaging

Plants

Seed stocks	No plants were used in this study.
Novel plant genotypes	No plants were used in this study.
Authentication	No plants were used in this study.
